# Treatment of intracranial inflammatory myofibroblastic tumor with PD-L1 inhibitor and novel oncolytic adenovirus Ad-TD-nsIL12: a case report and literature review

**DOI:** 10.3389/fimmu.2024.1427554

**Published:** 2024-07-24

**Authors:** Xiao Qian, Weihai Ning, Louisa Chard Dunmall, Yanming Qu, Yaohe Wang, Hongwei Zhang

**Affiliations:** ^1^ Department of Neurosurgery, Sanbo Brain Hospital, Capital Medical University, Beijing, China; ^2^ Centre for Cancer Biomarkers and Biotherapeutics, Barts Cancer Institute, Queen Mary University of London, London, United Kingdom

**Keywords:** intracranial inflammatory myofibroblastic tumor, immune checkpoints, oncolytic adenovirus, combination immunotherapy, case report

## Abstract

Inflammatory myofibroblastic tumor (IMT) is a rare pathological entity first described in 1939. This lesion is most commonly found in the lungs, but cases involving other systems, such as the central nervous system known as intracranial IMT (IIMT), have also been reported. Diagnosis currently relies on pathological results due to the lack of characteristic imaging changes. Surgical resection is an effective treatment, though the disease is invasive and may recur. Previous literature has reported a high level of programmed death 1 (PD-1) expression in IMT tissues, suggesting that immunotherapy may be effective for this condition. In this case report, we present a middle-aged male who received PD-1 inhibitor and oncolytic adenovirus (Ad-TD-nsIL12) treatment after IIMT resection surgery. This successful approach provides a new direction for the treatment of IIMT.

## Introduction

Inflammatory myofibroblastic tumor (IMT) is a rare disease, the pathogenesis of which is still unclear. Its pathological feature is the benign proliferation of inflammatory cells, and it is common in the lungs and upper respiratory tract (1). This disease can also rarely occur intracranially, as first reported by West SG and others in 1980 (2). Although, in most cases, the lesion is solitary, some studies have also reported multifocal involvement in intracranial and extracranial sites. The common symptoms of intracranial IMT (IIMT) are headache, epileptic seizures, ataxia, and visual impairment (3, 4). Magnetic resonance imaging (MRI) of the brain shows that the enhanced lesion is usually related to dura attachment, which is very similar to the neuroimaging manifestations of meningiomas. At present, the treatment of IIMT is more often surgery, supplemented by other treatments in the later stage (5). In previous studies, it has been reported that there is a large amount of programmed death 1 (PD-1) expression in IMT, which makes us think about the feasibility of immunotherapy in IMT (6). In this article, we report for the first time the use of the novel oncolytic virus (OV) Ad-TD-nsIL12 (7, 8) in combination with PD-1 inhibitors in a patient with IIMT.

## Case description

The patient is a 53-year-old man. Eight months before admission, he was admitted into another hospital for treating dizziness. A head MRI suggested a lesion in the left temporal region. A local biopsy was performed, and the pathology suggested IMT. Subsequently, a resection surgery was performed through a left postauricular incision. After the operation, the patient lost left ear hearing, had peripheral facial paralysis on the left side, choked when drinking water, and was discharged after the wound healed. The choking attenuated approximately 10 days after the operation, and the left facial paralysis recovered after more than a month. Two months before admission, the patient had no obvious cause of discomfort and swelling in the left temporal region, accompanied by dizziness, which was intermittent, occurring once every 2–3 days, each lasting approximately 10 min, and the pain could be gradually relieved. During the course of the disease, there was a brief loss of consciousness and tonic–clonic seizures of the limbs, which resolved after a few minutes. The head MRI suggested a recurrence of the left temporal mass, and the adjacent brain tissue was edematous. Later, he came to our hospital for further diagnosis and treatment. At the time of admission (referred to as day 0), the patient’s vital signs were stable, his mind was clear, the skin of the left mastoid was red and swollen, the pharyngeal reflex was slightly weakened, and the left ear was deaf. The head CT at the time of admission suggested that the left temporal tumor recurred. The soft tissue in the surgical area showed uneven thickening. The adjacent left maxillofacial skin was also unevenly thickened. The left part of the external auditory canal, tympanic wall, and ossicles was not clearly displayed. There was a bone defect in the posterior wall of the left mandibular joint. A large, slightly low-density lesion was observed near the left temporal lobe.

**Figure 1:**
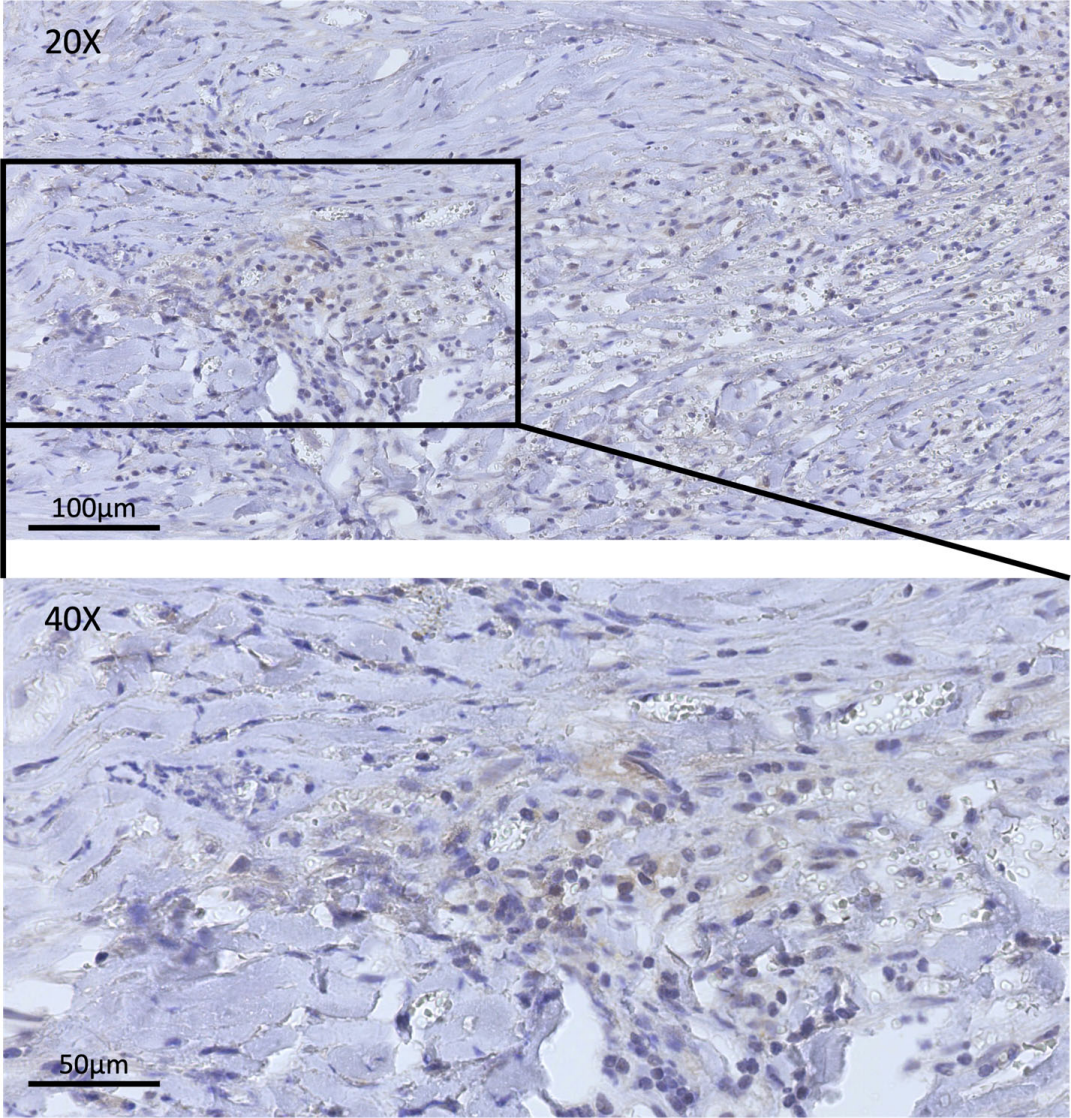
This figure shows the immunohistochemical staining of PD-L1 in samples achieved in surgery. In the pre-treatment tissue sample, positive staining for PD-L1 could be observed.

After the patient was admitted to the hospital, he completed the pre-operative examination and chose to have surgery. The former arc incision behind the left ear was used. During the operation, it was seen that the tumor tissue was filled in the mastoid bone defect area, which was gray-white, with moderate blood supply, and the boundary with the surrounding tissue was unclear. The tumor extended upward along the dura mater, which was significantly thickened. The tumor invaded the digastric muscle, splenius capitis, and lateral rectus capitis and other occipital and neck muscles downward and laterally, and the tumor invaded the lateral semicircular canal on the inside, and wrapped around the mastoid segment and tympanic segment of the facial nerve. During the operation, the tumor was near-totally removed, and at the same time, the diseased dura mater and muscle were totally removed. One milliliter of the original solution of Ad-TD-nsIL12 was taken (concentration 5 × 10^11^vp/mL), diluted to 5 mL with normal saline, and after being fully absorbed by gelatin sponge, it was attached to the dura mater surface and filled in the mastoid bone defect area, respectively. The postoperative pathology result reported as IMT and the immunohistochemical staining results indicated that PD-L1 was positive (**Figure 1**), which provided us with a basis for using inhibitors. The sigmoid sinus and facial nerve were well protected during the operation, and no embolism occurred after the operation. The postoperative facial nerve function was Grade III (House-Brackmann grading). After the operation, regular dehydration and hormone treatments were given. The patient recovered well and was discharged from the hospital.

**Figure 2:**
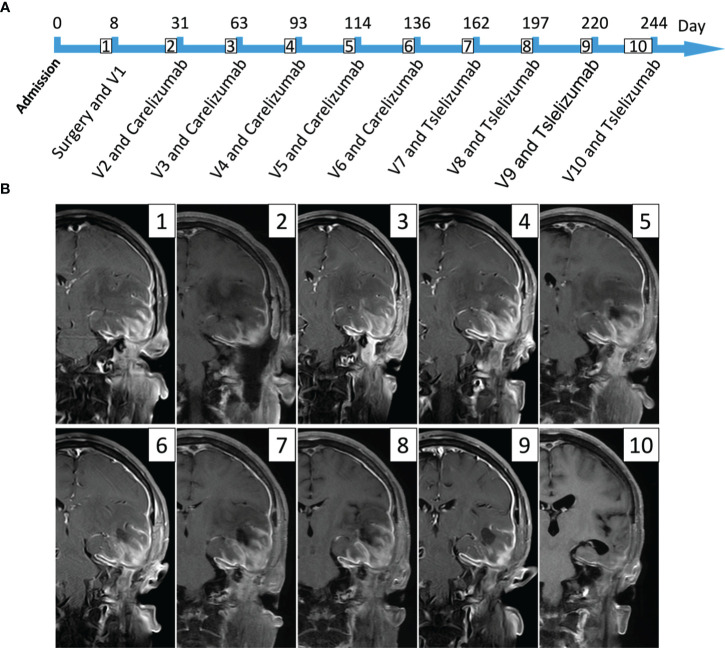
This figure mainly expresses the timeline of the patient’s treatment and the lesion changes in imaging. **(A)** The patient received a total of one intraoperative oncolytic virus treatment and then nine oncolytic virus and PD-1 inhibitor treatments. The first five times used 110 mg of carelizumab, and the last four times used 200 mg of tislelizumab; the dose of oncolytic virus used was 5 × 10^11^vp (V represents the number of virus treatments). **(B)** It mainly shows the changes in the patient’s MRI scans during treatment (T1 enhancement phase); the preoperative images (1) can see the tumor lesion, partly invading the surrounding soft tissues and dura mater; the left temporal lobe and insular edema are very severe. The postoperative MRI shows that the tumor in the left temporal region has been sub-totally removed, but the part that erodes the dura mater and surrounding soft tissues still exists. After using the combined treatment nine times, the last follow-up image (10) can hardly see the enhanced image, and the patient’s symptoms are almost completely relieved.

The patient subsequently underwent nine combined treatments of Ad-TD-nsIL12 + PD-1 inhibitor. The first five times used 110 mg of carelizumab 5 × 10^11^ vp + Ad-TD-nsIL12. Due to drug shortage, it was switched to 200 mg of tislelizumab + 5 × 10^11^ vp Ad-TD-nsIL12 in the subsequent four treatments. PD-1 inhibitors and Ad-TD-nsIL12 are used one after the other on two consecutive days, and the interval between each treatment is approximately 1 month (**Figure 2**). After the 5th and 7th injections of Ad-TD-nsIL12, the patient had epilepsy, all of which were minor attacks and relieved in 5 min. After the treatment, the pain and tissue swelling in the left temporal region of the patient gradually eased. On MRI scan, it can be seen that the enhancement of the dura mater and the surrounding soft tissue almost disappeared at the last treatment. The patient then stopped the combination treatment and was closely followed up. Two months after the last treatment, the patient had poor appetite, fatigue, and low blood pressure, fluctuating at approximately 81/55 mmHg and was treated at our hospital. To address cachexia, fluid replacement, gastrointestinal tube placement, and light amine were used to maintain blood pressure. Nasal feeding of nutritional fluid was given to sustain enteral nutrition. The patient’s albumin level gradually rose, and the cachexia improved. In the patient’s last follow-up, the patient’s condition was stable and no new symptoms appeared.

## Discussion

IMT is a rare mesenchymal tumor with moderate malignant potential (9). It was first discovered by Brunn in 1939. IMT was initially considered to be a reactive proliferation of certain benign diseases (10); however, in 2013, the World Health Organization (WHO) classified IMT as a bone and soft tissue tumor, with risks of local recurrence and distant metastasis of approximately 25% and 2%, respectively (11, 12). The disease is common in the lungs, mesentery, retina, and retroperitoneum of children, adolescents, and young people, and may also occur in the above systems (13, 14). At present, the pathogenesis of IMT is still unclear and may be related to various factors, such as EB virus infection, surgery, and trauma. In addition, the rearrangement and overexpression of anaplastic lymphoma kinase (ALK) gene on chromosome 2p23 have been proven to be closely related to the occurrence of IMT (4, 15). In 2000, Lawrence et al. found that TPM 3-ALK and TPM 4-ALK fusion genes are related to IMT (16). Since then, many ALK fusion genes have been reported, such as ATIC-ALK, CLTC-ALK, CARS-ALK, RANBP 2-ALK, and RRBP 1-ALK (17, 18). Therefore, ALK positivity helps in the diagnosis of IMT, but its absence cannot rule out the diagnosis of IMT, especially in adults (19). Therefore, the diagnosis of IMT still mostly relies on pathological results.

In addition to the common sites of IMT mentioned above, the disease can occur intracranially, and when it occurs intracranially, it can occur in almost all intracranial sites, among which the frontal lobe (23.6%) and temporal lobe (21.8%) are the main sites. The main symptoms of IIMT are headache (56.4%) and seizures (18.2%). There were a few cases of metastasis (3) and recurrence (10), with recurrences occurring as early as 6 months and as late as 11 years after treatment. Then, for the imaging of IIMT, the characteristics of tumors at different times seems to vary greatly; thus, it is often easily misdiagnosed. The disease that needs to be differentiated most is meningioma, because it has a very close MRI scan with meningioma, and there will be the appearance of meningeal tail sign. In addition, the MRI scan of IIMT may be distinguished significantly by the location of the lesion. There are reports of being misdiagnosed as acoustic neuroma in the cerebellopontine angle area (20). Most cases of IIMT MRI scan show equal or low signal on T1 WI and T2 WI, and diffusion-weighted imaging (DWI) usually shows a low signal. Most lesions will show enhancement of the lesion after using a contrast agent (5, 21). Treatments for IIMT include surgery, glucocorticoids, radiotherapy, chemotherapy, immunosuppressants, 6-thioguanine, methotrexate, NSAIDs, thalidomide, and atezolizumab. At the same time, it has been reported that IMT may have a certain self-limiting nature; that is, it will regress on its own during the course of the disease. Thus, the recent treatment of IIMT is still mainly surgery, supplemented by other treatments (22, 23).

Currently, research on immune checkpoints is becoming increasingly popular. Under normal circumstances, the immune system protects the host from autoimmune, allergic, and infectious diseases through a series of co-inhibitory and co-stimulatory receptors and their ligands (referred to as immune checkpoints) (24, 25). Increasing evidence suggests that tumors also use these mechanisms to evade immune responses and eventually progress, spread, and metastasize (25, 26). Among these pathways, PD-1 and the programmed cell death ligand 1 (PD-L1) axis play a key role in physiological immune homeostasis and have been widely studied (27). The binding of PD-L1 to its receptor inhibits T-cell migration, proliferation, and secretion of cytotoxic mediators, limiting tumor cell killing. PD-1 and PD-L1 inhibitors disrupt this axis, reversing T-cell inhibition and enhancing anti-tumor immunity, leading to long-term responses in patients with cancer. Additionally, PD-L1 interacts with B7 (CD80 and CD86), producing negative signals on T cells and inhibiting anti-tumor immunity (28, 29). The development and application of immune checkpoint inhibitors that block PD-1/PD-L1 interaction have resulted in very durable responses and prolonged the survival of various patients with cancer. The immunosuppressant of this target has shown clinical efficacy for many different solid and hematological malignancies. Research in soft tissue sarcomas suggests that the prevalence of PD-L1 expression varies between histological subtypes and may be an unfavorable prognostic feature, although this view is currently widely controversial (30–32). Two phase II clinical trials of pembrolizumab (anti-PD-1) treatment for advanced soft tissue sarcomas reported an objective remission rate of up to 18%, with a few patients with a PD-L1-positive tumor (33). When T cells are exhausted, they acquire multiple inhibitory molecules and cannot produce appropriate immune responses. Previous studies have confirmed that the expression rate of PD-L1 on tumors and infiltrating immune cells in IMT is high. In the evaluation of recurrent and metastatic IMT, 80% are PD-L1 (+). PD-L1 expression is also common in ALK (−) IMT (88%) (6). At the same time, 96% of PD-L1 (+) tumors show components of adaptive PD-L1 expression, which means that the disease is sensitive to PD-L1 treatment (34). In general, the current treatment of IMT using PD-1 inhibitors has a certain theoretical basis.

However, despite the emergence of ICIs that have completely changed cancer treatment, their best response rate when used alone is quite low, less than 35% to 40% (35–37). Part of the reason may be that although PD-1 inhibitors can improve the sensitivity of T cells in the microenvironment, they seem to have little effect on the transition from a cold environment to a hot environment (36). The emergence of OVs seems to have changed this situation. OVs are native or recombinant viruses targeting cancer cells. These viruses cause cancer cells to die at the end of the replication cycle by lysis or activating anti-tumor immune responses, thereby minimizing damage to normal tissues. At the same time, OVs are now considered effective immune stimulants, capable of activating and redirecting innate and adaptive immune responses against tumors. The goal of combining OVs and checkpoint inhibitors is to use viral infection to provide immune drive for tumors, change the local immune microenvironment into a more immunogenic environment, and make ICIs work more effectively in this environment (38–40). This combination has shown initial effectiveness in preclinical trials; thus, there are currently multiple clinical trials trying to combine the two to try to improve the anti-tumor efficacy of PD-1 inhibitors (40–42). Multiple adenovirus structures are undergoing preclinical testing with ICIs (43); some promising candidates include the recent report by the Hemminki group. In this study, they described two adenoviruses: one expressing tumor necrosis factor α (TNFα), and the other expressing IL-2. In their *in vivo* experiments with melanoma tumors established on the side of mice, they showed that when virus treatment was combined with the delivery of anti-PD-1 antibodies, the number of CD8^+^ T cells in the tumor significantly increased (compared to the virus alone). In addition, compared with virus monotherapy, the combination of PD-1 inhibitors and virus therapy resulted in significant tumor growth inhibition and promoted survival. A clinical trial is about to be conducted, in which the virus encodes TNF-α and IL-2 (TILT-123) in combination with anti-PD-1 antibodies.

The OV used in this case report is the third-generation oncolytic adenovirus (AdV) Ad-TD-nsIL12, which is a new generation of replicating adenovirus Ad-TD with three gene deletions (E1A CR2, E1B19K, and E3gp19K). The complete E3B region is retained to overcome the limitations of previous AdV candidate drugs (7). In addition to relying on the oncolytic effect and immune induction effect of the OV itself, the virus carries what is currently considered the most effective anti-tumor factor in the immune system, interleukin-12 (IL-12). However, despite the strong anti-tumor effect of IL-12, its significant toxicity in treatment has hindered its clinical application (7, 44, 45). In order to overcome the toxicity associated with IL-12 expression and take advantage of its strong anti-tumor activity, the virus carries a non-secretory IL-12 (nsIL12) molecule created by modifying the signal peptide of the cytokine. nsIL12 can achieve persistent, low-level expression of IL-12 in the TME (8). When expressed in Ad-TD, unmodified IL-12 is toxic after systemic administration, but modified IL-12 (nsIL12) is safe. It has been proven in preclinical studies to have significant anti-tumor effects on various solid tumors (7).

In this case of IIMT patients, we tried the combination of OV and PD-1 inhibitor treatment after surgery and achieved very good results. After the ninth course of treatment, not only did the patient’s tumor entity almost completely disappear, but the enhancement lesions on the infected dura mater and surrounding soft tissues almost disappeared as well. The patient reported that the pain in the left temple disappeared and the patient’s symptoms have not recurred in the last follow-up. This is a good outcome of combined treatment with OV and PD-1, which gives us confidence in the prospects of combined treatment with two drugs in subsequent medical development.

## Data availability statement

The original contributions presented in the study are included in the article/**Supplementary Material**. Further inquiries can be directed to the corresponding authors.

## Ethics statement

Written informed consent was obtained from the individual(s) for the publication of any potentially identifiable images or data included in this article.

## Author contributions

XQ: Writing – original draft, Writing – review & editing. WN: Writing – original draft, Writing – review & editing. LD: Writing – original draft, Writing – review & editing. YQ: Writing – original draft, Writing – review & editing. YW: Writing – original draft, Writing – review & editing. HZ: Writing – original draft, Writing – review & editing.
